# Research on digital empowerment, innovation vitality and manufacturing supply chain resilience mechanism

**DOI:** 10.1371/journal.pone.0316183

**Published:** 2025-02-12

**Authors:** Huiyan Wang, You Chen, Jiaping Xie, Caixuan Liu

**Affiliations:** 1 Xinjiang University of Finance and Economics, Urumqi, Xinjiang, China; 2 Shanghai University, Shanghai, China; 3 Shanghai University of Finance and Economics, Shanghai, China; 4 University of Wisconsin Madison, Madison, Wisconsin, United States of America; University of Almeria: Universidad de Almeria, SPAIN

## Abstract

As global manufacturing competition increasingly emphasizes supply chain resilience, enhancing the risk resistance of manufacturing supply chains through digital empowerment has become a critical priority. This study leverages the opportunities of the digital economy to deeply investigate the mechanisms that enhance supply chain resilience. Using data from China’ s A-share listed manufacturing companies from 2012 to 2020, a research framework is constructed based on information asymmetry and transaction cost theories. Employing text analysis and factor analysis, the study develops indicators for digital empowerment and supply chain resilience and examines their relationship through both theoretical analysis and empirical testing. The findings reveal that: (a) Digital empowerment significantly enhances supply chain resilience in the manufacturing sector, and this conclusion is robust across various robustness checks. (b) Mechanism analysis demonstrates that digital empowerment drives supply chain resilience primarily by enhancing innovation vitality within enterprises. (c) The moderating analysis shows that environmental uncertainty positively influences the resilience of digitally empowered manufacturing supply chains. (d) Further analysis indicates that the effects of digital empowerment on supply chain resilience vary depending on factor intensity, supply chain position, and industry competition levels. These results validate the positive role of digital empowerment in promoting supply chain resilience and explore the ’black box’ mechanism from the perspective of innovation vitality. The study also highlights the moderating influence of environmental uncertainty. By advancing the understanding of how the digital economy fosters high-quality development in manufacturing, this research provides actionable insights for strengthening supply chain resilience, achieving greater control over supply chain dynamics, and promoting deeper integration between digital technologies and the real economy.

## Introduction

The world is currently undergoing a profound transformation unprecedented in a century. Economic globalization is facing mounting resistance, while the international economic and political landscape is experiencing significant adjustments. The global supply chain is undergoing restructuring, with far-reaching implications for the supply chains of various countries. The effects of numerous emergencies have further exacerbated the "bottlenecks" and "shortcomings" in the supply chains of developing countries, constraining industrial development and impeding economic circulation.

On one hand, the outbreak of sudden events such as the COVID-19 pandemic and the Russia-Ukraine conflict has created significant concerns and anxieties among upstream, midstream, and downstream manufacturing enterprises about their survival and development, severely hindering the progress of manufacturing industries worldwide. On the other hand, the competition for dominance over industrial and supply chains has intensified, with developed countries employing political and economic measures to promote reshoring, "friend-shoring," and "near-shoring," further impacting the resilience of supply chains in developing countries [1]. In the context of a grid-based division of labor in global industries, the risk of supply chain disruptions poses a substantial threat to the transformation and sustainable development of manufacturing industries in developing countries.

With the slowdown of globalization and the rise of trade protectionism, the vulnerability of supply chains has become increasingly pronounced. As uncertainties in the global political and economic environment grow, external factors such as trade disputes and geopolitical conflicts have increasingly destabilized global supply chains, especially affecting manufacturing industries in developing countries. These external shocks exacerbate risks of industrial chain ruptures, supply chain control, and value chain blockages [2]. Although digital empowerment holds significant potential to enhance supply chain resilience, its effectiveness is often constrained by external factors, including regulatory restrictions, fragmented technical standards, and challenges related to data privacy and security [3]. In certain cases, the application and integration of digital technologies face obstacles from inconsistent standards and regulatory barriers. Therefore, fully harnessing the role of digital empowerment in strengthening supply chain resilience amid a volatile and uncertain external environment remains a critical challenge.

With the deepening of globalization and the accelerated evolution of digital empowerment, the competitive landscape has shifted from individual enterprises to chain network competition in the industrial and supply chains. The advantages of digital empowerment, such as data traceability [4], irreplaceability, and enhanced transparency [5], can effectively compensate for the defects in the supply chain, thereby enhancing its resilience and reducing the risk of chain breakage. Traditional supply chains, lacking digital technology support, often result in information islands, preventing timely and effective risk prediction and perception. This leaves supply chain enterprises facing high operational risks, slow market responses, and low collaboration efficiency. The integration of digital technology and the real economy can effectively improve the efficiency of information interaction and transparency among manufacturing enterprises.

For example, Lenovo Group has implemented a blockchain-supply chain integration model, enabling real-time information sharing among suppliers, enterprises, and foundries. Similarly, Walmart has adopted blockchain technology in its food supply chain using the IBM PaaS cloud platform, allowing for rapid traceability of food-related issues and enhancing resilience against supply chain risks [6].

Digital empowerment leverages advanced digital technologies to integrate enterprises, industries, and sectors into a cohesive network, transcending geographical boundaries and enhancing the scope and scale of knowledge spillovers for supply chain participants [7]. This integration not only enhances supply chain efficiency but also provides enterprises with expanded access to R&D resources, thereby fostering greater innovation capacity. On one hand, digital empowerment strengthens collaboration between supply chain participants (suppliers, partners, customers, etc.), thereby enhancing both market and environmental resilience in supporting enterprise innovation [8]. On the other hand, digital empowerment facilitates knowledge interaction between enterprises, universities, and research institutions [9], increasing the success rate of innovation and reducing R&D costs [10], thus strengthening supply chain resilience. Therefore, digital empowerment is becoming the driving force behind increasing R&D efficiency and supply chain resilience in the manufacturing industry. Its highly dependent and integrative nature provides crucial elements and environmental support for the formation of supply chain resilience.

Existing literature has predominantly emphasized the broad benefits and applications of digital empowerment, such as enhancing supply chain agility [11], developing supply chain financial platforms [12], and increasing supply chain transparency [13]. Most existing studies focus on the macro or industry-level effects of digital empowerment, with limited exploration at the enterprise level. Specifically, there is a lack of research on how digital technologies influence supply chain resilience through the interaction between internal mechanisms and external environments. To address these gaps, this paper utilizes data from China’s A-share manufacturing companies spanning 2012 to 2020. By employing text analysis and factor analysis, it constructs digital empowerment and supply chain resilience indicators and conducts empirical testing. The study aims to explore how digital empowerment enhances manufacturing supply chain resilience based on transaction cost and information asymmetry theories. Furthermore, R&D investment is introduced as a mediating factor to uncover the internal mechanisms at play. The moderating role of environmental uncertainty in the relationship between digital empowerment and supply chain resilience is also examined, contributing to the development of a novel research framework in this domain. By elucidating the "black box" of how digital empowerment impacts supply chain resilience, this research enriches current understanding of how the digital economy drives high-quality manufacturing development. Moreover, it offers actionable strategies for strengthening supply chain resilience and regaining control over supply chains in an increasingly uncertain global environment.

Relative to existing scholarship, this paper offers three key innovations and marginal contributions.

First, there is a scarcity of research on the resilience of China’s manufacturing supply chains. This paper constructs a research framework grounded in information asymmetry and transaction cost theories. Through empirical analysis, the study demonstrates that digital empowerment significantly enhances manufacturing supply chain resilience, with R&D investment serving as a mediating role in this process. Additionally, the impact of digital empowerment on supply chain resilience is moderated by environmental uncertainty.

Second, this paper overcomes the limitations of previous research methods. While existing studies on supply chain resilience have predominantly relied on surveys and theoretical analyses, this study identifies key factors that substantively influence supply chain resilience through a comprehensive literature review. Building on these insights, it constructs a supply chain resilience index for manufacturing firms based on micro-level data from listed companies. Using factor analysis, the index incorporates five dimensions: redundant resources, financial strength, collaboration relationships, operational capacity, and human capital. This approach broadens the methodological toolkit for measuring supply chain resilience.

Third, by elucidating the mechanisms through which digital empowerment enhances manufacturing supply chain resilience, this paper contributes to accelerating the transformation and upgrading of the manufacturing industry, stabilizing the industrial, supply, and value chains. Using China as a case study, this research offers important policy insights and practical pathways for advancing manufacturing modernization in developing countries and fostering the integration of the digital economy with the real economy.

## Literature review and research hypotheses

### Literature review on digital empowerment supply chain resilience

With the rapid advancement of digital technologies such as the Internet of Things, Big Data, and 5G, the global economy has undergone a digital transformation, and traditional supply chains have transitioned into the digital era. Supply chain resilience, as an emerging field in supply chain management, has garnered increasing attention. The term "resilience" has long been a subject of study in disciplines such as psychology and ecosystems. However, a unified explanation or definition of supply chain resilience has not yet been established. Christopher and Peck were among the first to define supply chain resilience as the ability of a supply chain system to restore to its initial state after an operational disruption, encompassing flexibility, adaptability, and agility [14]. The most comprehensive definition of supply chain resilience comes from Ponomarov and Holcomb, who define it as the ability of a supply chain to adapt to sudden unforeseen events and disruptions, maintain structural and functional stability, and recover from shocks [15]. They further categorize supply chain resilience into four dimensions: readiness, response, recovery, and growth. Specifically, building supply chain resilience requires capacity building in pre-crisis risk prevention, rapid response to disruptions, and quick recovery and growth after risks materialize [16].

From an enterprise perspective, supply chain resilience is a multi-dimensional capability, with its core objective being the sustainable development of companies. In the digital era, digital empowerment holds significant potential to integrate supply chain management [17], thereby enhancing both supply chain resilience and sustainable development [18]. For example, Wang et al. found that under the impetus of China’s “dual carbon” goals, some high-tech retail enterprises have achieved green transformation through digital technologies, thereby enhancing their supply chain resilience. By analyzing these enterprises’ cases, it was revealed that digital empowerment not only improved operational efficiency but also fostered greater environmental awareness, which positively impacted their financial performance [19]. Additionally, Xue et al. highlighted that within big data pilot zones, digital innovation has enabled enterprises to optimize energy utilization efficiency, thereby enhancing the sustainability of their supply chains. This practice not only improves enterprises’ ability to respond to unexpected events but also provides long-term development assurance [20]. While supply chain resilience has garnered widespread attention, there remains a critical need to explore how digital empowerment can reinforce supply chain resilience across various dimensions and advance its development. Drawing on existing research, this paper categorizes the relationship between digital empowerment and supply chain resilience into the following dimensions:

(1) Collaboration capability perspective: The collaboration capability of supply chain subjects participants enhance collaboration efficiency based on the digital supply chain service platform [21], fostering overall synergy between the upstream and downstream elements of the supply chain and thereby strengthening supply chain resilience [22]. Digitalized supply chains require enterprises to transition from stability to adaptability, from standardization to customization, and from isolation to collaboration [23]. For instance, blockchain, as a peer-to-peer distributed network, can effectively mitigates information asymmetry within the supply chain and promote collaboration among its participants [24]. Saglam et al. highlighted the critical role of the coordination among supply chain members to realize supply chain resilience, and from the three dimensions of the quality of communication, reciprocity, and relational commitment, they emphasized that the process and systematic collaboration among supply chain members is a very important part of supply chain resilience. Collaborative relationships at both the process and system levels among supply chain members are integral to the development of supply chain resilience [25].

(2) Operational capability perspective: One of the specific manifestations of supply chain disruption is operational disruption, which significantly impacts supply chain resilience [26]. Digital empowerment enables modern organizations to leverage hardware, software, and communication networks to facilitate the development of platform-based supply chains [27], and the emergence of platforms and services enhance enterprises’ operational capabilities across purchasing, production, and sales processes. Zhu et al. highlighted that digital empowerment allows modern organizations to enhance operational capabilities in purchasing, production, and sales through the use of hardware, software, and communication networks [28]. Additionally, Fu et al. found that information accessibility significantly impacts enterprise operations in a digitalized environment, emphasizing the importance of information flow in enhancing operational capabilities [2]. Brusset and Teller conducted a questionnaire survey on 177 managers based on the resource-based view based on the theory of dynamic capabilities, and concluded that supply chain resilience is fundamentally an operational capability that enables interrupted supply chains to recover and strengthens supply chain resilience [29]. Therefore, digital empowerment not only enhances the operational capabilities of individual enterprises but also promotes synergies across the entire supply chain, strengthening its ability to address disruption risks.

(3) Financial strength perspective: Supply chain resilience is significantly and positively associated with corporate financial performance in the event of risky shocks [30]. The emergence of digital supply chain platforms provides dual credit services to supply chain enterprises, improving accessibility and affordability, enhancing demand responsiveness, and mitigating the risk of supply chain disruptions. This also helps address the financing challenges faced by micro, small, and medium-sized enterprises (MSMEs) in the supply chain [31]. Chen et al. examined the relationship between corporate financial performance and environmental performance in the context of a low-carbon economy, highlighting that digital transformation can enhance financial flexibility, enabling enterprises to better manage risks [32]. Kaur et al. emphasized that digital empowerment enables supply chain participants to respond rapidly and accurately to demand, providing services that satisfy stakeholders and making these services accessible and affordable, thus improving the financial strength of supply chain participants [33]. Based on the theory of resource orchestration, Jamal et al. verified the positive relationship between supply chain resilience and enterprise financial performance through the analysis of a structural equation model of 289 French firms [34].

(4) Redundant resource perspective: During state of supply chain disruption, supply chain members holding a higher inventory share will have a more favorable cost advantage, mitigating the shock of supply chain disruption as much as possible [35]. Lin et al. observed that current optimization of redundant resources in enterprises focuses primarily on inventory, which significantly shortens product life cycles and reduces cash flow velocity. However, the digitization of traditional supply chains can transform traditional inventory control and management approaches, breaking down barriers between enterprises and modern logistics [36]. Leung et al., utilizing a digital twin framework, integrated machine learning and simulation to optimize inventory replenishment policies, thereby minimizing the cash conversion cycle of the supply chain [37]. A digital supply chain platform that integrates simulation, machine learning and optimization enable real-time monitoring and analysis of key metrics such as production and inventory, enhancing service efficiency and reducing total costs during risky shocks. In supply chain risk management, business operations such as safety stock, maintenance of multiple suppliers, and low capacity utilization can be achieved to mitigate the impact of supply chain disruptions by keeping resources in reserve, which in turn improves the ability to anticipate and adapt to risks [38].

(5) Human capital perspective: The relational and human capital possessed by supply chain managers facilitates the establishment of key prerequisites for supply chain resilience, such as visibility, responsiveness, and flexibility, which collectively in turn strengthens supply chain resilience [39]. Schrauf et al. argued that the effective development of digital supply chains depends on the awareness of supply chain participants and the digital competence of employees [40]. The digital competence of employees in supply chain enterprises is a key factor in driving the advancement of digital supply chain, and the digital competence of employees will be transformed into enterprise knowledge stock, which promotes the formation of supply chain resilience through mutual learning among supply chain participants [41].

**Fig 1:**
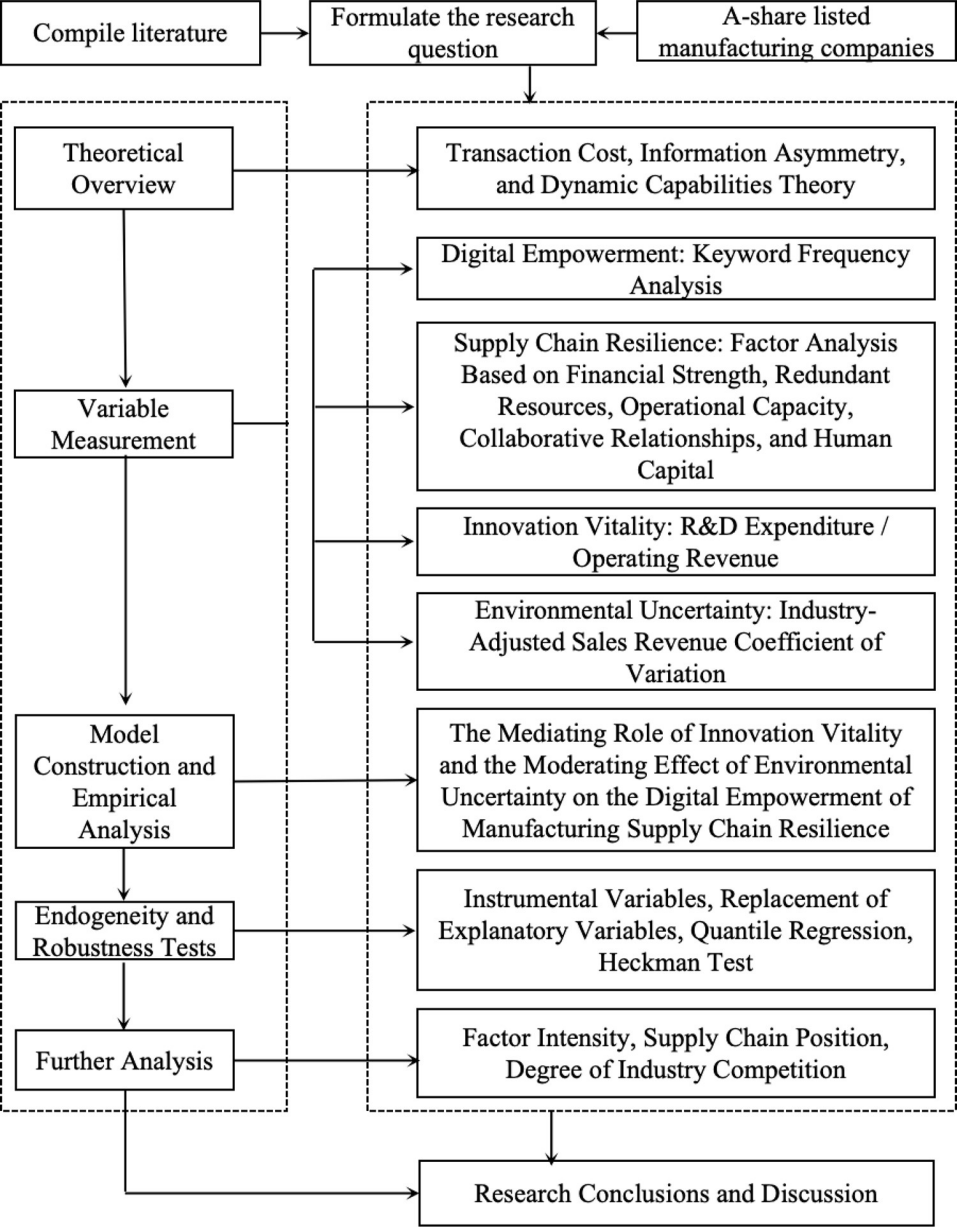
Research framework.

Through the above research, it is evident that there are multiple perspectives in the existing research on supply chain resilience, indicating that supply chain resilience is a complex set of concepts and that digital empowerment of supply chain resilience is not isolated or unilateral but is rooted in multi-dimensional factors, including collaborative relationships, operational capabilities, financial strength, redundant resources, and human capital. In today’s industrial landscape characterized by division of labor and specialization, the impact and influence faced by the manufacturing supply chain will also show complexity and depth. Therefore, focusing solely on the digital empowerment of the supply chain is insufficient. Instead, a comprehensive multi-dimensional assessment of how to enhance the resilience of the manufacturing supply chain in the context of digitization is particularly important. However, to date, the existing literature has not considered the supply chain resilience of digitally-enabled manufacturing supply chains from a multi-dimensional perspective. This paper aims to measure supply chain resilience based on the aforementioned five dimensions in order to explore the ’black box’ mechanism of digitally-enabled manufacturing supply chain resilience. The specific research framework is shown in Fig 1.

### Research hypotheses

#### Digital empowerment of manufacturing supply chain resilience

In the turbulent business environment, enterprise digital empowerment will become an effective means to strengthen supply chain resilience, and its impact is primarily reflected in the following aspects:

(1) Digitalization can effectively alleviate the degree of information asymmetry between upstream and downstream enterprises in the supply chain. In the traditional supply chain, there are many information silos, resulting in supply and demand imbalance and other problems. The adoption of digital technologies accelerates the speed of information dissemination and exchange between upstream and downstream enterprises. Leveraging characteristics such as memory and traceability inherent in digital elements, enterprises can more accurately predict changes in user demand [42]. This shift promotes a transition in inventory management decision-making from a reactive ’induction-response’ model to a proactive ’forecast-execution’ model, enabling to better integrate internal and external resources and fully harness the potential of digitalization to supplement, strengthen, and stabilize supply chains. Therefore, digitalization improves the visualization of supply chain information by breaking down information silos, breaks the vertical linear structure of the traditional supply chain, improving responsiveness to market changes through structural adaptation. This makes supply chain decision-making more intuitive [43], allowing for better management of uncertainty, more accurate predictions, and proactive measures, including early prevention, early detection and early preparation. prevention, early detection, early preparation, reduce the risk of chain breakage, and enhance supply chain resilience.

(2) Digitization can effectively reduce external transaction costs, thereby enhancing supply chain resilience. According to Goldfarb and Tucker’s research [44], the cost reduction of digitalization for the supply chain is mainly reflected in the digital empowerment to generate modern logistics to reduce logistics costs, as well as to strengthen corporate trust to reduce the high costs caused by default risk. First of all, for the manufacturing industry, the traditional logistics and transportation methods, there are low efficiency, transportation costs and other supply chain management issues, with the application of digital technology, through the intelligent logistics, cloud computing, big data and other information technology for warehousing optimization, fusion of warehousing network and distribution network, greatly enhance the efficiency of logistics, and at the same time the application of digitalization also guarantees the security of logistics delivery, reduce the enterprise information collection cost and information verification cost. Secondly, the interests of all parties in the supply chain are inconsistent, and there are motives to transfer false information to upstream and downstream enterprises for the sake of profit. The permanent, traceable and unchangeable characteristics of blockchain data ensure the accuracy and authenticity of information transfer between enterprises, while government departments can continuously monitor this data, enabling preemptive issue identification and the establishment of effective regulatory mechanism [45]. Based on the characteristics of blockchain data, multiple subjects involved in the supply chain facilitates cooperation among each other, reduce unnecessary inventory, rationalize production planning, and thus effectively reduce production costs. The reduction of supply chain costs will directly affect regional commodity prices, stimulate market demand, and strengthen the market influence of enterprises. The cost reduction will promote the dynamic balance between supply and demand, make the enterprises in the supply chain accelerate the integration of the industry chain, avoid the migration of manufacturers in the chain, reduce the redundancy of enterprises, increase the stickiness of enterprises, anticipate the external risk, and make the exchange of resources between the enterprises in the supply chain more convenient and more resilient [46].

In summary, the application of digitalization for the supply chain is comprehensive and multifaceted. By alleviating the ’information silo’ and cost loss phenomena among supply chain enterprises, digitalization significantly enhances redundant resources, financial performance, collaborative relationships, and operational capacity. Simultaneously, the reduction of supply chain costs prompted enterprises to encourages enterprises to actively invest more resources in further building a modernized supply chain. At the same time, the reduction of supply chain costs prompts enterprises to actively invest more resources to further build a modernized supply chain, enhance inter-enterprise adhesion, gain market competitiveness to prevent key enterprises from leaving the supply chain, and further alleviate the crisis of supply chain breakage in the face of unknown risks. Based on this, the first hypothesis is proposed.

H1: Firms’ digital empowerment can significantly improve supply chain resilience.

#### Mediating role of innovation dynamics

R&D and innovation are usually regarded as key factors for enterprises to improve their competitiveness and realize transformation and upgrading. However, the R&D and innovation process is fraught with uncertainties regarding technological and market prospects. These uncertainties lead to unpredictable outcomes, requiring enterprises to make substantial, sustained, and stable capital investments. For most enterprises, the risks associated with innovation are difficult to bear, resulting in reduced motivation to pursue R&D and innovation, thereby diminishing the vitality of enterprise R&D activities [47]. The enterprise digital empowerment can alleviate this phenomenon partially. Digital empowerment can effectively alleviate the degree of information asymmetry, thus reducing the uncertainty of R&D innovation. First, digital technologies offer unparalleled advantages in information generation, storage, and transmission. These capabilities enhance factor synergy and reduce market failures caused by information asymmetry [48]. Moreover, the permanence, traceability, and immutability of blockchain data further alleviate information asymmetry both within and between enterprises, thereby improving resource allocation, enhancing R&D efficiency, and encouraging enterprises to increase investment in innovation [49]. Second, digital empowerment addresses information asymmetry while also influencing interactions between financial institutions and enterprises [50]. The application of digital technology to enhance the ability of financial institutions to reveal information will also compelling enterprises to improve the quality of information disclosure. This improvement increases the availability of long-term credit, enabling enterprises to secure more stable financial resources. Consequently, digital empowerment promotes growth in innovation inputs by fostering corporate information transparency and improving access to long-term credit resources [51].

In a supply chain environment, firms may face various risks originating from supply, process, demand, control, and the external environment, resulting in potential disruptions within the supply chain in which each organization operates [52], and firms’ innovation inputs, as a dynamic organizational capability, influence supply chain resilience from multiple perspectives [53]. First, through corporate digital empowerment, corporate innovation behaviors will enhance firms’ ability to identify changes in the external environment and establish appropriate action plans to address risks [54]. Second, corporate innovation behaviors generate cutting-edge designs and solutions, enabling firms to respond effectively to unforeseen environmental changes and risk shocks thereby preventing chain-breaking crises. Third, the innovative behavior of enterprises enhances market competitiveness, which is accompanied by faster responses to market changes and gradual evolutionary improvements [55]. This also implies that firms’ ability to identify external risks increases over time, helping to prevent supply chain disruptions and improve resilience. Therefore, enterprises that allocate sufficient resources to innovation, experience lower exit risks, longer survival periods, and stronger resilience against supply chain crises [56]. Based on this, this paper proposes the second hypothesis.

H2: Firms’ digital empowerment can enhance supply chain resilience by promoting the growth of innovation dynamics.

#### The moderating role of environmental uncertainty

In the era of digital economy, supply chains and the enterprises in the chain are operate in highly uncertain environments. The rules of international trade are undergoing significant changes, with FTAs, higher customs unions, and common markets increasingly dominating global trade regulations, making uncertainty the norm. According to Teece’s theory of dynamic capabilities, ’dynamic’ refers to the ability to adjust promptly to changes in the external environment, while ’capability’ denotes the capacity to build, integrate, and reorganize resources [57]. Supply chain resilience is the scenario of coping with external shocks in a volatile and unpredictable environment, therefore, environmental uncertainty in the process of digitally empowering supply chain resilience is bound to have a deep impression on the formation of supply chain resilience.

Environmental uncertainty can affect supply chain resilience at both micro and macro levels. At the micro level, supply chain uncertainty primarily consists of demand uncertainty, supply uncertainty and technological uncertainty. First, demand uncertainty can lead to unknown and unpredictable demand quantity and time in the supply chain, resulting in inventory surpluses or shortages [58]. In turbulent environments, digital supply chain platforms can foster cooperative relationships among supply chain enterprises, enabling them to access necessary resources from partners, maintain market competitiveness, and collectively resist supply chain risks [59]. Secondly, supply uncertainty can lead to production stoppages, quality problems, logistical obstacles, etc. Walker and Weber pointed out that due to the opportunistic behavior of suppliers, they will change suppliers, adjust production and product specifications in the face of supply uncertainty, which will lead to unnecessary cost changes [60]. Therefore, digital platforms enhance collaboration between enterprises, strengthening the supply chain’s capacity to withstand risks associated with supply uncertainty. Finally, technological uncertainty requires firms to be able to quickly predict and share information to maintain their innovation capability in a state of rapid technological change. In turbulent environments, stronger supply chain relationships, facilitated by digital empowerment, enable enterprises to share information efficiently and enhance supply chain resilience. From the macro level, environmental uncertainty is mainly manifested in economic policy uncertainty [61], governments frequently adjust economic and trade policies to stabilize growth and smooth fluctuations, making it difficult for enterprises to anticipate changes in the policy environment. So enhancing supply chain resilience with digital empowerment becomes an important means for enterprises to cope with environmental uncertainty [62]. Based on this, this paper proposes the third hypothesis.

H3: Environmental uncertainty has a positive moderating effect on supply chain resilience in digitally empowered manufacturing.

**Fig 2:**
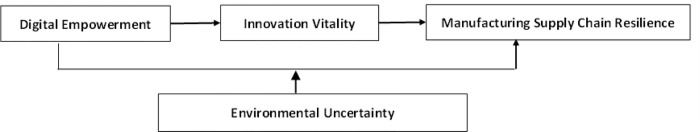
Construct model.

Based on the above theoretical derivation and research hypotheses, the following Fig 2 conceptual model is constructed:

## Research design

### Sample selection and data sources

Given the rapid development of digital technologies in China and their integration with the real economy primarily since 2012, as well as to ensure the availability of digital empowerment data, this study selects China’s A-share manufacturing firms listed from 2012 to 2020 as the initial research sample. This period avoids the significant disruptions caused by the COVID-19 pandemic in 2021–2022, which resulted in supply chain breaks and delays for many firms, creating unavoidable shocks to manufacturing supply chains from both market and policy perspectives. The data handling process follows these steps: first, companies labeled as ST, PT, or those insolvent are excluded; second, instances with significant missing key data are omitted; and third, to mitigate the effects of data outliers, a 1% bilateral Winsorization is applied to continuous variables. The primary data source is the CSMAR database.

The explanatory variables (supply chain resilience), explanatory variables (digital empowerment), mediating variables, moderating variables, and control variables are discussed below:

(1) Supply chain resilience (Scr): the above literature review and analysis indicate that this paper constructs dimensions through five categories: redundant resources, financial strength, collaborative relationships, operational capacity, and human capital. These dimensions encompass a total of seven quantitative indicators, specific indicators as detailed in Table 1, and are measured using factor analysis. The specific measurement process is described in the following section.

(a) Redundant resources: Following methodologies employed in prior research, the ratio of management expenses to operating income is selected as an indicator to measure an enterprise’s redundant resources [63].

(b) Financial strength: As a critical measure of an enterprise’s capacity, financial strength is included as a key dimension of supply chain resilience. This paper uses return on net assets and net profit margin on total assets as indicators to assess financial strength [64].

(c) Collaboration: Based on a comparison of existing studies, this paper selects supply chain concentration as the indicator of collaboration [65].

(d) Operating capacity: Drawing on prior research results, accounts payable turnover and accounts receivable turnover are chosen as indicators to evaluate operating capacity [66].

(e) Human capital: Existing studies commonly use the ratio of employees with bachelor’s degree or higher as the indicator of human capital [67].

**Table 1:** Definition and measurement of variables.

Variable types and dimensions		Variable name	Variables and measurement
Explained variable	Financial strength	Return on net assets(ROE)	Net profit/shareholders’ equity balance (X1)
		Total Assets Net Profit(ROA)	Net profit/total assets balance (X2)
	Redundant resources	Sinking redundant resources(RS)	Administrative expenses/operating income (X3)
	Operational capacity	Accounts payable turnover(Aptr)	Operating costs/average accounts payable occupancy (X4)
		Accounts receivable turnover ratio(Rtr)	Operating income/average occupancy of accounts receivable (X5)
	Collaborative relationships	Supply chain concentration(Scc)	Average of the sum of the ratio of sales to purchases from the top 5 suppliers and customers (X6)
	Human Resource	Percentage of people with a bachelor’s degree or higher(Degree)	Number of persons with bachelor’s degree and above as a ratio of the total number of persons with bachelor’s degree and above (X7)
Explanatory variable		Digital Empowerment (Digi)	ln(1+Total number of keywords on digitization)
Mediating Variable		Innovation Vitality (R&D)	R&D investment/revenue
Moderator variable		Environmental Uncertainty (Eu)	Industry-adjusted coefficient of variation of sales revenue
Control Variable		Cash Flow (Cashflow)	Net profit/total assets balance
		Growth (Growth)	(Total assets at the end of the current period -total assets at the end of the previous period)/total assets at the end of the previous period
		Top 1 Shareholding (Top1)	Ratio of the number of shares held by the largest shareholder of the enterprise to the total number of shares of the enterprise
		Enterprise size (Size)	Logarithm of total assets at the end of the period
		Liability (Lev)	Total assets/total liabilities

(2) Digital Empowerment (Digi): Digital empowerment is a theoretical concept that has emerged alongside the development of digital technologies. At its core, it refers to "empowerment through digital technologies." Most scholars define it as the use of digital technologies to optimize organizational structures, business models, and management systems, thereby enhancing organizations’ knowledge accumulation and skill levels [68]. Enterprise digital empowerment is a systematic process, and existing research on digitalization measures mainly approaches it from the following perspectives.

(a) Some studies use the proportion of intangible assets and IT investment to measure the density of enterprise informatization [69, 70]. However, the level of enterprise informatization investment does not necessarily reflect the level of its actual application [71].

(b) The other part of the literature uses a questionnaire survey to measure the degree of digital transformation of the enterprise by adopting the proportion of enterprise information technology personnel [72], yet the use of computers may just be a simple web application behavior. Unlike the above research literature, this paper draws on Wu Fei et al.’s study [73] and uses textual analysis to portray the digital empowerment of enterprises.

Since enterprises often outline digital initiatives as part of their strategic development, these are reflected in their annual reports through summaries and guidance. The vocabulary used in these reports reflects the company’s future strategies, business philosophy, and development trajectory. Moreover, the vocabulary usage in the annual report can reflect the future strategy of the enterprise as well as its business philosophy and development path. Therefore, it is feasible to use text analysis to count the word frequency in the annual reports of enterprises. The specific steps are as follows: First step, annual reports of A-share manufacturing firms from 2012 to 2020 are collected. Python is used to extract the ’business analysis’ sections from these reports. The second step, identify digital empowerment keywords by reviewing national digitization-related policies and research reports issued between 2012 and 2020, such as the "Special Action Plan for Digital Empowerment of SMEs," the "2020 Digital Transformation Trend Report," and the "China Digital Transformation Research Report (2022)," as well as government work reports and research publications by the China Academy of Information and Communication Technology. Building on the research of Wu Fei et al. [73], the scope of digital empowerment feature words is further expanded to form a keyword library, covering AI, Big Data, Cloud Computing, Blockchain, and digital applications. In the third step, the annual reports are textually analyzed. Based on the digital empowerment thesaurus obtained in the second step, Python software is used to capture the digital empowerment keywords in the annual reports. In the fourth step, Words with negative modifiers (e.g., ’no’ or ’none’) preceding keywords are excluded. Keyword frequencies are counted, categorized, and totaled. To address the right-skewed nature of the data, keyword frequencies are logarithmized to create the digital empowerment index.

(3) Mediating and moderating variables:

(a) Mediating variable: Innovation vitality, measured by the ratio of R&D investment to business revenue in the manufacturing industry.

(b) Moderating variable (Eu): Environmental uncertainty, which can be narrowly defined as institutional factors such as policies and laws, or more broadly as encompassing macroeconomic, institutional, and physical environments. In enterprise-level studies, environmental uncertainty is often defined as a comprehensive external environment that affects business operations and can lead to fluctuations in business performance. With reference to the method of Shen Huihui et al. [74], environmental uncertainty is measured using the industry-adjusted coefficient of variation of business revenue. Since environmental uncertainty originates from fluctuations in external environments that affect an enterprise’s business activities, it ultimately leads to fluctuations in sales revenue. Its specific calculation formula is as follows:

$$Sale=\theta_{0}+\theta_{1}Year+\varepsilon$$ (1)

In Eq (1), Sale represents firm’s sales revenue, Year denotes the year variable (Year = 1 for observations in the forth year, Year = 2 for the third year, and so on, up to Year = 5). The residuals obtained from the OLS regression of Eq (1) are the abnormal sales revenues, which are obtained by dividing the standard deviation of the abnormal sales revenues obtained in the 5 years by the average of the sales revenues in the 5 years. unadjusted industry uncertainty. Finally, the industry-adjusted environmental uncertainty indicator is obtained by dividing the industry-adjusted environmental uncertainty by the median environmental uncertainty of the industry in which the sample is located.

(4) Control variables: In order to ensure the accuracy of the study, a total of five control variables are selected in this paper:

(a) Enterprise size (Size): The data in this paper is selected from the data of A-share listed companies. Measured by the natural logarithm of total assets at the end of the previous period, based on the existing digitized literature;

(b) Cash flow (Cashflow): To capture the trend of corporate cash in the future, measured by the balance of net profit and total assets;

(c) Growth(growth): Capturing the development status and development speed of the enterprise’s business capability, measured by the ratio of total assets in the current period to total assets in the previous period;

(d)Liability (Lev): Captures the ability of a company to conduct business activities through creditor funds and the level of corporate liabilities, measured by the ratio of total assets to total liabilities;

(e) Top 1 Shareholding (Top1): Reflects corporate governance by capturing the level of ownership concentration. It is measured by the ratio of shares held by the top shareholder to the firm’s total shares.

### Treatment of supply chain resilience variables

#### Applicability and correlation test

The applicability and correlation test is conducted using the KMO test, which compares the correlation coefficient and partial correlation coefficient between variables. The results of the KMO test and Bartlett’s test, obtained through SPSS software, are presented in Table 2. From Table 2, it can be observed that the KMO statistics are all greater than 0.5, and the P-values of Bartlett’s sphericity test are all 0.000. These results indicate that the selected variables in this study have strong correlations with each other and are suitable for factor analysis.

**Table 2:** Results of KMO test and Bartlett test for cross-sectional data from 2012 to 2020.

Year		2012	2013	2014	2015	2016	2017	2018	2019	2020
KMO		0.514	0.527	0.521	0.524	0.521	0.535	0.563	0.573	0.582
Bartlett’s spherical test	Approximate chi-square	2266.589	2284.922	2279.808	2332.449	2868.684	3744.416	4449.661	5069.394	5173.712
	df	21	21	21	21	21	21	21	21	21
	Sig.	0	0	0	0	0	0	0	0	0

#### Factor analysis

Since some original data points are missing, which could affect the results of factor analysis, this paper employs the linear interpolation method to address the missing values. Specifically, a linear function is constructed based on the neighboring samples of the missing values to ensure sufficient sample size. Additionally, a shrinking-tail treatment is applied to mitigate the impact of extreme values. The principal component method is used to extract the common factors, and the eigenvalues and variance contribution rates of these factors are presented in Table 3. The eigenvalues of the first three factors are all greater than 1, with a cumulative variance contribution rate of approximately 65%. Thus, the three extracted factors effectively capture the information of the secondary indicators.

**Table 3:** Contribution rate of total variance in factor analysis of cross-section data from 2012 to 2020.

Year	Component	Initial eigenvalue			Sum of squares loading extraction			Rotated sum of squares loading		
		Total	Variance%	Cumulative%	Total	Variance%	Cumulative%	Total	Variance%	Cumulative%
2012	1	1.912	27.316	27.316	1.912	27.316	27.316	1.911	27.307	27.307
	2	1.631	23.296	50.612	1.631	23.296	50.612	1.627	23.246	50.553
	3	1.053	15.039	65.651	1.053	15.039	65.651	1.057	15.098	65.651
2013	1	1.956	27.940	27.940	1.956	27.940	27.940	1.915	27.362	27.362
	2	1.584	22.622	50.561	1.584	22.622	50.561	1.602	22.879	50.241
	3	1.054	15.057	65.619	1.054	15.057	65.619	1.076	15.378	65.619
2014	1	1.916	27.370	27.370	1.916	27.370	27.370	1.910	27.279	27.279
	2	1.593	22.760	50.130	1.593	22.760	50.130	1.348	19.254	46.533
	3	1.077	15.379	65.509	1.077	15.379	65.509	1.328	18.976	65.509
2015	1	1.958	27.977	27.977	1.958	27.977	27.977	1.942	27.748	27.748
	2	1.563	22.329	50.306	1.563	22.329	50.306	1.308	18.683	46.430
	3	1.035	14.789	65.095	1.035	14.789	65.095	1.307	18.665	65.095
2016	1	1.978	28.261	28.261	1.978	28.261	28.261	1.875	26.785	26.785
	2	1.454	20.769	49.030	1.454	20.769	49.030	1.547	22.101	48.886
	3	1.046	14.936	63.966	1.046	14.936	63.966	1.056	15.080	63.966
2017	1	2.121	30.307	30.307	2.121	30.307	30.307	1.907	27.236	27.236
	2	1.437	20.525	50.831	1.437	20.525	50.831	1.635	23.355	50.592
	3	1.039	14.848	65.679	1.039	14.848	65.679	1.056	15.087	65.679
2018	1	2.197	31.388	31.388	2.197	31.388	31.388	2.042	29.174	29.174
	2	1.325	18.932	50.320	1.325	18.932	50.320	1.451	20.723	49.897
	3	1.011	14.437	64.756	1.011	14.437	64.756	1.040	14.860	64.756
2019	1	2.244	32.057	32.057	2.244	32.057	32.057	2.125	30.358	30.358
	2	1.311	18.728	50.786	1.311	18.728	50.786	1.423	20.328	50.686
	3	1.034	14.771	65.557	1.034	14.771	65.557	1.041	14.870	65.557
2020	1	2.229	31.849	31.849	2.229	31.849	31.849	2.126	30.368	30.368
	2	1.317	18.811	50.660	1.317	18.811	50.660	1.416	20.226	50.594
	3	1.026	14.653	65.313	1.026	14.653	65.313	1.030	14.719	65.313

#### Synthesis of the composite index

The scores of the three factors (F1,F2,F3), and the coefficient matrix of the 2019 factor scores are presented in Table 4, and the factor scores of a sample company are calculated based on the coefficient matrix of the 2019 factor scores:

F1 = 0.895X1+0.897X2-0.660X3+0.311X4+0.253X5-0.071X6-0.151X7

F2 = 0.311X1+0.321X2+0.219X3-0.670X4-0.512X5-0.073X6+0.595X7

F3 = 0.103X1+0.080X2+0.197X3+0.188X4-0.006X5+0.954X6+0.153X7

**Table 4:** Factor score coefficients in 2019.

Secondary indicator	Component			Secondary indicator	Component		
	1	2	3		1	2	3
Return on net assets(X1)	0.895	0.311	0.103	Accounts receivable turnover ratio(X5)	0.253	-0.512	-0.006
Total Assets Net Profit(X2)	0.897	0.321	0.080	Supply chain Concentration(X6)	-0.071	-0.073	0.954
Sinking redundant resources(X3)	-0.660	0.219	0.197	Percentage of people with a bachelor’s degree or higher(X7)	-0.151	0.595	0.153
Accounts payable turnover(X4)	0.311	-0.670	0.188				

Subsequently, based on the variance contribution rate of each public factor, the composite factor score (Scr) of supply chain resilience of the sample company is derived. Taking 2019 as an example, the Scr of a sample company is calculated as follows:

Scr=(32.057%×F1+18.728%×F2+14.771%×F3)/65.557%

Using the same method, the composite index score of supply chain resilience (Scr) is calculated sequentially for each listed company across all years, providing a consistent measure of supply chain resilience.

### Descriptive statistics

The results of the descriptive statistics are presented in Table 5, with a total of 14,163 samples obtained through data organization and matching. The mean value of supply chain resilience (Scr) is 7.3876 with a standard deviation is 6.894, indicating significant variation in supply chain resilience among individuals. For digital empowerment (Digi), the mean and standard deviation are 1.2419 and 1.275, respectively, with minimum and maximum values of 0 and 6.06. These results suggest a noticeable disparity in the level of digital empowerment among enterprises, reflecting an uneven integration of digitalization and the real economy. Additionally, the horizontal differences among the other variables are significant, and the variability of each control variable is large. All main variables fall within a reasonable range, highlighting the substantial gap in digital empowerment levels among listed companies. Notably, some enterprises have yet to enhance their supply chain resilience through digital empowerment. Therefore, testing the supply chain resilience of China’s manufacturing industry in the context of digital empowerment holds strong theoretical and practical significance.

**Table 5:** Descriptive statistics.

Variable	Sample size	Mean	std	Min	Median	Max
Scr	14163	7.3876	6.894	-38.61	6.63	152.22
Digi	14163	1.2419	1.275	0.00	1.10	6.06
Cashflow	14163	0.0525	0.065	-0.20	0.05	0.26
Growth	14163	0.1536	0.367	-0.73	0.10	4.81
Top1	14163	0.3358	0.140	0.08	0.32	0.76
Size	14163	22.0611	1.154	19.52	21.90	26.40
Lev	14163	0.3896	0.188	0.03	0.38	0.98
R&D	14163	4.7546	5.417	-3.43	3.81	304.15
Eu	14163	1.3569	1.420	0.06	0.96	15.25

### Model setting

Based on the hypotheses proposed in this paper, the following regression model is constructed to examine the impact of digital empowerment on manufacturing supply chain resilience:

$${Scr}_{i,t}=\alpha +\beta {Digi}_{i,i}+\gamma {Controls}_{i,t}+\varepsilon_{it}$$ (2)

In Eq (2), the explanatory variable Scr represents supply chain resilience, as previously defined. The explanatory variable Digi denotes the digital transformation of enterprises, and the coefficient β measures the extent of Digi’s influence on supply chain resilience. Controls is the firm-level control variable. Finally, in order to control the impact of year and industry factors on supply chain toughness, this paper adds industry and year fixed effects in the equation.

To further explore the mechanisms through which digital empowerment influences manufacturing supply chain resilience, the following model is constructed based on Eq (2) using enterprise innovation vitality as the mediating variable.

$${R\&D}_{i,t}=\alpha_{1}+\eta_{1}{Digi}_{i,t}+\eta_{2}{Controls}_{i,t}+\varepsilon_{it}$$ (3)

$${Scr}_{i,t}=\alpha_{2}+\gamma_{1}{Digi}_{i,i}+\gamma_{2}{R\&D}_{i,t}+\gamma_{3}{Controls}_{i,t}+\varepsilon_{it}$$ (4)

In this model, R&D serves as the mediating variable, representing innovation vitality, while other variables remain the same as in Eq (2). A significant regression coefficient β indicates that R&D contributes to the mediating mechanism of digital empowerment in enhancing supply chain resilience, although it may not be the sole mechanism.

In order to analyze whether environmental uncertainty moderates the relationship between digital empowerment and supply chain resilience, Eq (2) incorporates the interaction term between environmental uncertainty and digital empowerment:

$${Scr}_{i,t}=\alpha_{1}+\beta_{1}(Digi*Eu)+\beta_{2}{Digi}_{i,i}+\beta_{3}{Eu}_{i,t}+\gamma_{1}{Controls}_{i,t}+\varepsilon_{it}$$ (5)

Here, Eu denotes environmental uncertainty, and the coefficient of the interaction term represents the moderating effect of environmental uncertainty on the relationship between digital empowerment and supply chain resilience.

## Empirical results and analysis

### Benchmark regression

Table 6 presents the regression results for digital empowerment(Digi) and supply chain resilience (Scr). The regression coefficients of Digi, the primary focus of this paper, are all significantly positive across columns (1) (2) (3). In column (1), which includes control variables, the regression coefficient is 0.173 and significant at the 1% level. Column (2) incorporates industry and year fixed effects, resulting in a regression coefficient of 0.111, significant at the 5% level. In column (3), after controlling for both industry and year fixed effects, the regression coefficient increases to 0.224, remaining significant at the 1% level. These findings tentatively suggest that digital empowerment effectively enhances manufacturing supply chain resilience, supporting the validity of Hypothesis 1.

**Table 6:** Empirical results of digital empowerment of manufacturing supply chain resilience.

	(1)	(2)	(2)
	Scr	Scr	Scr
Digi	0.173***	0.111**	0.224***
	(3.37)	(2.43)	(4.32)
Cashflow		-2.068**	-1.590*
		(-2.23)	(-1.78)
Growth		0.551***	0.818***
		(3.49)	(5.46)
Top1		0.695*	0.0745
		(1.66)	(0.19)
Size		-0.259***	-0.264***
		(-4.30)	(-4.44)
Lev		-1.141***	-1.318***
		(-3.09)	(-3.71)
_cons	7.173***	13.20***	13.38***
	(85.89)	(10.66)	(10.90)
Industry	Yes	No	Yes
Year	Yes	No	Yes
N	14163	14163	14163
R2	0.132	0.00547	0.137

Note: Numbers in parentheses are t-values

***, **, * indicate significant at 1%, 5%, and 10% confidence levels, respectively.

### Mechanism path and moderating effect tests

#### Mechanism path test

To verify hypothesis 2, this paper uses innovation vitality as the mechanism variable, and further explores the influence mechanism of digital empowerment on manufacturing supply resilience based on the steps of mediation effect test.

Column (3) of Table 7 indicates that digital empowerment can effectively promote the growth of manufacturing innovation vitality and improve the innovation level of supply chain enterprises, with a regression coefficient of 0.349, which is significant at the 1% level. In column (1), which excludes control variables, the results remain significant (coefficient 0.259, p<0.01). Column (4) reports the test results of the mediating effect of firms’ innovation vitality (coefficient 0.185, p<0.01). Similarly, in column (2), which excludes control variables, the coefficient is 0.141, also significant at the 1% level. These findings suggest that digital empowerment contributes to the resilience of manufacturing supply chains by promoting increased innovation vitality among firms in the supply chain, thereby supporting Hypothesis 2.

**Table 7:** Test results of mechanism path and regulatory effect.

	(1)	(2)	(3)	(4)	(5)
	R&D	SCR	R&D	SCR	SCR
Digi	0.259***	0.141***	0.349***	0.185***	0.131**
	(6.46)	(2.77)	(8.73)	(3.57)	(1.98)
R&D		0.122***		0.111***	
		(11.35)		(10.23)	
Digi*Eu					0.0698**
					(2.27)
Eu					0.0466
					(0.84)
Cashflow			-6.260***	-0.894	-1.171
			(-9.09)	(-1.00)	(-1.30)
Growth			-0.196*	0.840***	0.752***
			(-1.70)	(5.63)	(4.96)
Top1			-1.702***	0.264	0.120
			(-5.49)	(0.66)	(0.30)
Size			-0.287***	-0.232***	-0.199***
			(-6.26)	(-3.92)	(-3.14)
Lev			-3.803***	-0.895**	-1.214***
			(-13.87)	(-2.51)	(-3.40)
_cons	4.433***	6.634***	13.06***	11.93***	11.82***
	(67.77)	(69.31)	(13.79)	(9.68)	(8.74)
Industry	Yes	Yes	Yes	Yes	Yes
Year	Yes	Yes	Yes	Yes	Yes
N	14163	14163	14163	14163	14163
R2	0.137	0.139	0.169	0.144	0.138

Note: Numbers in parentheses are t-values

***, **, * indicate significant at 1%, 5%, and 10% confidence levels, respectively.

#### Moderating effect test

To verify Hypothesis 3, Eq (2) is expanded by including the moderator variable (Eu) and the interaction term between the independent variable (Digi) and the moderator variable (Digi × Eu). This allows for testing the moderating effect of environmental uncertainty (Eu) on the relationship between digital empowerment (Digi) and supply chain resilience (Scr). Column (5) of Table 7 presents the specific test results for the moderating effect. The coefficient of the interaction term, which is the primary focus of this analysis, the regression coefficient of 0.0698, at the 5 percent level significantly positive. This result demonstrates that environmental uncertainty exerts a significant moderating effect on supply chain resilience of digitally empowered manufacturing industry, and the dynamic and evolving external environment will contribute to supply chain resilience of digitally empowered supply chain, hypothesis 2 is proved. Meanwhile, the regression coefficient of Digi is 0.131, which is significant at 5% level, again corroborates the conclusion of hypothesis 1.

### Endogeneity and robustness test

Troubleshooting endogeneity. Selecting appropriate instrumental variables is an effective way to solve the endogeneity problem. Drawing on the methodology of Huang Qunhui et al. [75], this study uses the number of landline telephones per million people in 1984 in historical cities is used as an instrumental variable for digital empowerment. On the one hand, dial-up access to telephone lines marked that the Internet began to gradually come into the public’s view, followed by ISDN, ADSL, and fiber-optic broadband access, and at the same time, contributed significantly to the continued development of the subsequent digital technology. On the other hand, along with the development of digital technology and the economy, the frequency of the use of traditional dial-up telephones has gradually declined in line with the exclusivity. In addition, since the original data of instrumental variables are cross-sectional data, this study adopts the approach of Nunn and Qian [76] by incorporating dynamic variables. Specifically, the amount of national Internet investment and the number of landline telephones per million people in the preceding year are used to construct interaction terms, enabling the instrumental variable for digital empowerment to vary over time.

From the results in column (1) (2) of Table 8, it can be seen that the F-test for the instrumental variables exceeds the critical value for the 10% level of the Stock-Yogo weak identification test. Additionally, the P-value of the LM statistic is 0.000, which significantly rejects the original hypothesis. These findings confirm the validity of the selected instrumental variables. Furthermore, the regression results for the impact of digital empowerment on manufacturing supply chain resilience remain significantly positive after addressing endogeneity concerns, thereby reaffirming the validity of Hypothesis 1.

**Table 8:** Endogeneity and robustness tests.

	(1)	(2)	(3)	(4)
VARIABLES	First stage	Second stage	Scr	Scr
IV	0.089***			
	(6.25)			
Digi		5.299***	0.236***	0.0302***
		(4.15)	(4.52)	(4.18)
Cashflow	-0.344**	0.205	3.520*	-1.529*
	(-2.37)	(0.17)	(1.93)	(-1.71)
Growth	0.057**	0.530**	-2.181**	0.839***
	(2.35)	(2.57)	(-2.31)	(5.60)
Top1	-0.057	0.293	0.949***	0.162
	(-0.88)	(0.56)	(5.77)	(0.40)
Size	0.177***	-1.186***	0.0881	-0.285***
	(18.60)	(-4.86)	(0.22)	(-4.71)
Lev	-0.182***	-0.295	0.195	-1.285***
	(-3.15)	(-0.56)	(0.80)	(-3.61)
Constant	-4.634***	40.187***	-2.180***	12.98***
	(-8.25)	(5.97)	(-3.82)	(10.63)
LM Statistic	39.177			
	[0.000]			
F Statistic	39.045			
	{16.38}			
Industry	Yes	Yes	Yes	Yes
Year	Yes	Yes	Yes	Yes
N	14,163	14,163	14,163	14,163
R2	0.336	-0.449	0.138	0.137

Note: Numbers in parentheses are t-values

***, **, and * indicate significance at 1%, 5%, and 10% confidence levels, respectively, [] values are P-values, and {} values are critical values at the 10% level of the Stock-Yogo weak identification test.

Heckman two-step method. To address the endogeneity caused by sample selection bias in the model, the Heckman two-step model is employed. In the first stage, a digital empowerment dummy variable (Digi_dum) is constructed using the annual median of the digital empowerment variable (Digi_m). Values greater than or equal to the median are assigned 1, while others are assigned 0. A Probit regression is then performed with the digital empowerment dummy variable, Digi_m, and the control variables from the baseline regression, and the inverse Mills ratio (Imr) is calculated. In the second stage, the Imr is incorporated into Eq (2) for regression. As reported in column (3) of Table 8, the estimated coefficient of Digi is 0.236, significantly positive at the 1% level. Additionally, the estimated coefficient of Imr passes the 10% significance level, indicating the presence of sample selection bias in this study. After adjusting for this bias, the research conclusions remain robust.

Controlling for the effect caused by industry year events. To address potential interference from industry- and year-specific events, regional-year interaction fixed effects are introduced in addition to the industry and year fixed effects. This approach accounts for region-specific time effects and mitigates the impact of special regional changes on the baseline regression. As reported in column (3) of Table 8, the coefficient of Digi is 0.240, which is significantly positive at the 1% level.

Replacement Variable Tests. To further ensure the robustness of the findings, the digital transformation index is employed as an alternative for the digital empowerment index, and the replacement variable is reintroduced into Eq (2) for further testing. The digital transformation index is derived from the CSMAR (Cathay Pacific) database, and is computed by assigning weights of 0.3472, 0.162, 0.0969, 0.0342, 0.2713, and 0.0884 to six key indicators: strategic leadership, technology-driven, organizational empowerment, environmental support, digital outcomes, and digital applications. These values are then aggregated to produce the final digital transformation index. As reported in column 4 of Table 8, the Digi coefficient is 0.0302, which remains significantly positive at the 1% level.

Quantile regression. Quantile regression was employed to exam in the differential impact of digital empowerment across varying levels of supply chain resilience. Quantile regression can capture the effects of the independent variable under different conditions, making it particularly well-suited for analyzing heterogeneous impacts at different levels. Based on Eq (2), Table 9 presents the results of quantile regression at the 10%, 30%, 50%, 70%, and 90% quantiles of supply chain resilience. The findings reveal that digital empowerment significantly promotes supply chain resilience at all levels, and the stronger the supply chain resilience, the greater the effect of digital empowerment on it.

**Table 9:** Quantile regression.

	(1)	(2)	(3)	(4)	(5)
	q-10	q-30	q-50	q-70	q-90
Digi	0.0993**	0.143***	0.165***	0.197***	0.307***
	(2.11)	(5.21)	(5.57)	(4.90)	(3.71)
Cashflow	-4.738***	-4.102***	-4.120***	-3.821***	-1.108
	(-5.84)	(-8.66)	(-8.05)	(-5.50)	(-0.78)
Growth	0.457***	0.462***	0.453***	0.610***	0.681***
	(3.36)	(5.82)	(5.28)	(5.23)	(2.84)
Top1	-0.0224	-0.0835	0.155	0.190	0.768
	(-0.06)	(-0.39)	(0.67)	(0.61)	(1.20)
Size	-0.270***	-0.236***	-0.243***	-0.274***	-0.246***
	(-5.01)	(-7.47)	(-7.13)	(-5.94)	(-2.59)
Lev	-0.619*	-0.916***	-1.306***	-1.586***	-2.124***
	(-1.92)	(-4.86)	(-6.41)	(-5.74)	(-3.74)
_cons	12.74***	13.10***	14.41***	16.28***	25.72***
	(4.11)	(7.24)	(7.37)	(6.14)	(4.71)
Industry	Yes	Yes	Yes	Yes	Yes
Year	Yes	Yes	Yes	Yes	Yes
N	14163	14163	14163	14163	14163

Note: Numbers in parentheses are t-values

***, **, * indicate significant at 1%, 5%, and 10% confidence levels, respectively.

## Further analysis

The above empirical results of this paper underscore that digital empowerment effectively fosters the enhancement of manufacturing supply chain resilience. This positive effect is achieved, in part, through the promotion of innovation vitality within supply chain enterprises. Moreover, the resilience-building effects of digital empowerment are moderated by environmental uncertainty, which enhances the formation of supply chain resilience in unstable external environments. Further, this paper explores the differences in the impact of digital empowerment on supply chain resilience from the perspective of factor intensity, upstream and downstream positioning, and the degree of industry competition.

(1) Factor Intensity Perspective. The manufacturing industry can be categorized into three types based on factor intensity: technology-intensive, capital-intensive, and labor-intensive industries. The development of labor-intensive industries predominantly relies on resource endowments and human capital. However, the current level of digital empowerment in China’s labor-intensive industries is relatively low compared to that of capital- and technology-intensive industries. Labor-intensive industries exhibit weak cohesion and low digital correlation indices [77]. In contrast, technology- and capital-intensive industries, characterized by robust research and development capabilities, substantial market demand, high growth potential, high knowledge intensity, and significant R&D investment, demonstrate an intrinsic need for and adaptability to digitalization. These industries benefit from accelerated transformation and upgrading processes, driving enterprise innovation and enhancing the resilience of manufacturing supply chains. Consequently, digital empowerment of capital-intensive manufacturing industries facilitates the rapid enhancement of supply chain resilience.

Following Zhao Chenyu’s methodology [78], enterprises with a fixed asset-to-total asset ratio greater than or equal to the median are classified as capital-intensive. Technology-intensive enterprises are identified based on a ratio of R&D expenditures to payable employee compensation exceeding 1, while the remaining enterprises are classified as labor-intensive. As reported in column (1) of Table 10, digital empowerment supply chain resilience has a significant enhances in capital-technology-intensive enterprises, with a regression coefficient of 0.245, which is significantly positive at the 1% level.

**Table 10:** Further analyses the test results.

	(1)	(2)	(3)	(4)	(5)	(6)
	capital and technology-intensive type	labor-intensive type	downstream	upstream	low-competitive industry	highly competitive industry
Digi	0.245***	0.125	0.0271	0.351***	0.366***	0.0791
	(4.85)	(0.40)	(0.39)	(4.77)	(5.24)	(1.07)
Cashflow	-2.595***	3.090	-0.962	-1.907	-1.498	-0.775
	(-2.92)	(0.71)	(-0.85)	(-1.45)	(-1.20)	(-0.62)
Growth	0.712***	1.205*	0.573***	1.068***	0.312	1.242***
	(4.78)	(1.68)	(3.21)	(4.59)	(1.60)	(5.67)
Top1	0.235	0.00823	0.535	-0.312	0.636	-0.234
	(0.60)	(0.00)	(1.04)	(-0.53)	(1.15)	(-0.42)
Size	-0.226***	-0.586*	-0.257***	-0.277***	-0.414***	-0.183**
	(-3.85)	(-1.86)	(-3.39)	(-3.19)	(-5.00)	(-2.22)
Lev	-1.564***	0.720	-1.369***	-1.308**	-1.418***	-0.990*
	(-4.47)	(0.37)	(-3.12)	(-2.45)	(-2.98)	(-1.95)
_cons	12.65***	19.30***	13.09***	13.73***	16.66***	11.48***
	(10.42)	(2.95)	(8.35)	(7.65)	(9.71)	(6.75)
Industry	Yes	Yes	Yes	Yes	Yes	Yes
Year	Yes	Yes	Yes	Yes	Yes	Yes
N	13133	1030	6318	7845	6188	7975
R2	0.142	0.203	0.133	0.148	0.148	0.156

Note: Numbers in parentheses are t-values

***, **, * indicate significant at 1%, 5%, and 10% confidence levels, respectively.

(2) Supply Chain Upstream and Downstream Location Perspective. This paper examines the differential impact of digital empowerment on upstream and downstream supply chain resilience, emphasizing the importance of distinguishing supply chain locations to accurately identify weak links under risk impact. The current phenomenon of ’digital hegemony’ in some countries predominantly targets control over upstream segments of the digital supply chain. In response, the advancement of China’s digital empowerment initiatives has accelerated the establishment of an autonomous and controllable digital product supply chain [79]. For the domestic manufacturing sector, upstream intermediate products are sourced through both global and domestic supply chain channels. However, global supply chains are more susceptible to disruptions caused by risks such as wars and natural disasters. In contrast, domestic supply chains demonstrate greater stability and stronger self-regulation capabilities [80]. Leveraging digital supply chain platforms can effectively reduce information search costs and support the construction of multi-location supply chain systems. Such systems enhance the stability of upstream supply chains, mitigate the risks associated with digital hegemony, and enable timely utilization of alternatives when disruptions occur.

In this paper, the method of Antrà et al. [81] is employed to measure the upstream degree of the industry in which the enterprise operates. The median upstream degree for each year is used as the differentiation point. Enterprises with an upstream degree greater than or equal to the median are classified as upstream enterprises of the supply chain, while the remaining samples are categorized as downstream enterprises. The regression results, reported in columns (3) and (4) of Table 10, indicate that the promotional effect of digital empowerment on the resilience of upstream supply chains in China’s manufacturing industry is more pronounced. Specifically, the regression coefficient is 0.351 and is significantly positive at the 1% level.

(3) Industry Competition Degree Perspective. The external environment represented by industry competition, increasingly influences the development of supply chains and their enterprises. On one hand, excessive industry competition not only amplifies the complexity of enterprise decision-making but also weakens the competitiveness of the enterprise’s product market [82]. It compresses the time and space for resource acquisition, reduces profits, and increases operational pressure, which can trigger an internal cohort effect within the industry, ultimately impacting the overall stability of the supply chain [83]. On the other hand, in a less competitive industry environment, upstream and downstream supply chain relationships tend to be more stable. Enterprises in such environments focus more on product quality, long-term competitive advantages, and the overall development of the supply chain. This stability benefits the consolidation of supply chain resilience and enhances its capacity to resist risks.

In this study, the industry competition index is constructed based on the industry Herfindahl index. The sample is categorized into strongly competitive and weakly competitive industries according to the median industry competition degree. The regression results, presented in columns (5) and (6) of Table 10, indicate that a weakly competitive industry environment more effectively fosters supply chain resilience in digitally empowered manufacturing industries. Specifically, the regression coefficient is 0.366 and is significantly positive at the 1% level.

## Conclusion and insights

The rapid digitization of the global economy, driven by digital technologies such as the Internet of Things (IoT), big data, and 5G, has ushered traditional supply chains into the digital era. The strategic integration of these digital technologies into traditional supply chains, fostering the creation of digital supply chains, has become a focal point. This transformation aims to strengthen supply chain resilience and enhance the risk mitigation capabilities of manufacturing supply chains, serving as a cornerstone for the high-quality development of China’s manufacturing industry. Employing data from China’s A-share listed manufacturing companies spanning the years 2012 to 2020, this study employs text mining techniques and factor analysis to construct indicators for digital empowerment and supply chain resilience, respectively. The investigation delves into the role of digital empowerment in the mechanism of manufacturing supply chain resilience. The findings suggest:

(1) Digital empowerment significantly enhances manufacturing supply chain resilience.

(2) The augmentation of enterprise innovation vitality serves as a critical pathway through which digital empowerment reinforces manufacturing supply chain resilience.

(3) Environmental uncertainty positively moderates the relationship between digital empowerment and manufacturing supply chain resilience.

(4) Factors such as factor intensity, supply chain location, and industry competition exhibit differentiated effects on digitally empowered manufacturing supply chain resilience, influencing the robustness of the digital-enabled manufacturing supply chain. The implications of this study offer valuable policy insights for bolstering manufacturing supply chain resilience and accelerating the high-quality development of the manufacturing sector.

Management-Level Recommendations. At the management level, enterprise managers should strategically adapt to the transformation demands of the digital era by actively promoting digital empowerment. First, managers must prioritize the integration of digital technologies into supply chain management by formulating a comprehensive digital transformation strategy. This strategy should systematically advance the digitalization of the supply chain, ensuring that digital technologies are embedded across all stages—from production to supply and logistics—thereby enhancing efficiency throughout the entire process. Second, enterprise managers should increase investments in digital infrastructure, focusing particularly on advanced technologies such as the Internet of Things (IoT), big data, and artificial intelligence (AI). These technologies enable real-time monitoring, data-driven analysis, and intelligent decision-making within the supply chain, significantly strengthening the enterprise’s ability to predict and respond to risks. By leveraging these tools, enterprises can enhance their overall control and adaptability in an increasingly complex and dynamic business environment.

Enterprise-Level Recommendations. At the enterprise level, businesses should actively capitalize on the development opportunities brought about by digital empowerment. From a micro perspective of supply chain resilience, enterprises should leverage digital empowerment to strengthen their position within the supply chain and focus on building robust digital capabilities. Actively promoting the digital transformation of traditional manufacturing supply chains can significantly enhance their ability to resist risks. To this end, enterprises should continually improve their innovation capabilities by increasing investment in research and development (R&D). Through the additional attributes generated by enhanced innovation capacity, enterprises can better perceive external information, improve risk awareness, and respond to crises more effectively. Leveraging digital technologies also facilitates the flow of technology and funding across supply chains, stimulating industry innovation vitality and enhancing overall risk resistance. Labor-intensive enterprises, in particular, must adapt to the digital era by integrating digital technologies and attracting digital talent to accelerate their digital transformation. They should prioritize breaking down barriers in data connectivity with upstream and downstream enterprises, enabling smoother data flow across the supply chain. Furthermore, these enterprises should draw inspiration from capital- and technology-intensive counterparts by increasing the added value of their products and shifting focus toward higher-value segments of the supply chain. This strategic shift allows enterprises to align with industry realities and foster development through technological advancements, strengthening both the value chain and supply chain while bolstering their ability to withstand shocks. Additionally, enterprises should rationally design their digital development strategies based on factor intensity, external environmental changes, and the current state of industry competition. Such strategies should guide upstream and downstream enterprises toward collaborative development, ensuring the integration of production, supply, and sales functions. This approach comprehensively enhances supply chain resilience and its ability to mitigate risks in volatile environments. Enterprises can further utilize digital empowerment to strengthen collaborative relationships with supply chain partners, facilitating resource exchange and information sharing, which reinforces supply chain stability and adaptability in uncertain conditions.

Industry-Level Recommendations. At the industry level, achieving high-quality development in the manufacturing sector requires a dual focus: advancing the digitalization of individual supply chain enterprises and strengthening collaborative relationships between upstream and downstream participants. Leveraging data as a core element, enterprises should utilize the transmission effects of digital technology to enhance coordination and resilience across the entire supply chain. This process involves accelerating the integration of data channels between upstream and downstream enterprises, enabling smoother information flow and resource sharing. By employing digital technologies, such as big data and blockchain, enterprises can facilitate the efficient flow of technology and capital, fostering industry-wide innovation and enhancing overall risk resilience. For upstream supply chains, digital empowerment should be harnessed to stabilize the system by forming alliances through digital platforms. These alliances can significantly reduce information search costs, improve risk identification, and strengthen control mechanisms to mitigate vulnerabilities. Furthermore, enterprises should formulate digital strategies tailored to factors such as resource intensity, external environmental changes, and industry competition. These strategies must align with current industry conditions and foster the collaborative development of upstream and downstream enterprises. By promoting integrated production, supply, and marketing, enterprises can create a more cohesive and robust supply chain capable of withstanding risks and external shocks.

Governmental-Level Recommendations. At the governmental level, efforts should focus on accelerating the establishment and implementation of policies related to supply chain resilience while promoting the widespread deployment of digital supply chains. First, governments should evaluate the current market conditions across industries and foster the development of a group of representative digital supply chain enterprises. These core enterprises should act as leaders in enhancing supply chain resilience, setting examples with replicable practices that can guide both the industry and supply chain participants in collaboratively building highly resilient digital supply chains. Second, for industries and enterprises operating under diverse external conditions, tailored policies should be designed based on local contexts. Such policies should address specific challenges and bottlenecks that hinder the strengthening of supply chain resilience, ensuring that enterprises receive targeted support to overcome these obstacles effectively.

## Limitations and future outlook

The findings of this study have significant theoretical and practical implications for effectively leveraging digital resources, promoting their integration with the real economy, and advancing supply chain resilience from a micro-level perspective. However, certain objective limitations, primarily related to data availability, leave room for improvement and further exploration:

First, the measurement of enterprise digital empowerment in this study lacks a certain degree of precision. By employing Python-based text recognition technology, keywords related to the "digital economy" were extracted from the annual reports of listed companies to construct the digital empowerment index. While the study incorporated a substantial number of listed companies as the research sample, digital empowerment is also relevant to non-listed companies. Due to the absence of comprehensive data on non-listed enterprises in existing databases, these entities were excluded, potentially introducing selection bias into the sample. Future research should aim to refine the measurement methodology by adopting a broader definition of digital empowerment. Additionally, surveys could be conducted to directly assess the level of digital empowerment in companies, yielding more robust and persuasive results.

Second, the current methods for measuring supply chain resilience are predominantly reliant on questionnaire-based surveys, with few studies utilizing large-scale data from listed companies. Given that supply chain resilience is inherently multidimensional and complex, the reliance on specific variables may lead to selection bias or an incomplete assessment of a supply chain’s capacity to resist and recover from risks. Future research should consider integrating questionnaire-based approaches with large-sample datasets to achieve a more comprehensive and nuanced measurement of supply chain resilience.

Lastly, while this study examines the relationships among digital empowerment, R&D investment, supply chain resilience, and environmental uncertainty, new factors influencing these relationships are likely to emerge over time. The dynamic nature of digital technologies and supply chain systems warrants further exploration of additional variables and antecedents that may affect both digital empowerment and supply chain resilience. Future research should strive for a more holistic analysis, capturing the evolving landscape of factors shaping supply chain resilience and digital transformation.
